# Altered behavior in mice overexpressing soluble ST2

**DOI:** 10.1186/s13041-020-00606-4

**Published:** 2020-05-11

**Authors:** Motoshi Kikuchi, Kenkichi Takase, Morisada Hayakawa, Hiroko Hayakawa, Shin-ichi Tominaga, Tsukasa Ohmori

**Affiliations:** 1 Laboratory of Natural History, Jichi Medical University School of Medicine, Tochigi, Japan; 2 Laboratory of Psychology, Jichi Medical University School of Medicine, Tochigi, Japan; 3 Department of Biochemistry, Jichi Medical University School of Medicine, Tochigi, Japan; 4 Japan Association for Development of Community Medicine (JADECOM), 2-6-4 Hirakawacho, Chiyoda-ward, Tokyo, Japan

**Keywords:** Soluble ST2, Depression, Mouse, Forced swimming test, Tail suspension test

## Abstract

Psychoneuroimmunological studies have clearly demonstrated that both cellular and humoral immunity are related to major depression. Soluble ST2 is regarded as a key molecule regulating immune system as well as cell proliferation. Indeed, soluble ST2 is reported to reduce IL-33-induced IL-6 and TNF-α production in macrophages and IL-33-induced IL-5 and IL-13 production in type 2 innate lymphoid cells. Elevated serum concentrations of soluble ST2 have been reported in patients with neuropsychiatric disorders, suggesting pathophysiological roles of soluble ST2 in behavioral phenotypes. Nevertheless, the relation between soluble ST2 and depressive behavior remain to be uncovered. To complement this point, we performed broad behavioral phenotyping, utilizing transgenic mice with a high concentration of serum ST2 in the present study. Soluble ST2 overexpression mice (ST2 Tg mice) were generated on a C3H/HeJ background. ST2 Tg mice crossed onto the BALB/c genetic background were used. Before starting tests, each mouse was observed in a clean cage for a general health check and neurological screening tests. In Experiment I, comprehensive behavioral phenotyping was performed to reveal the role of soluble ST2 on sensorimotor functions, anxiety-like behaviors, depression-like behaviors, social behaviors, and learning and memory functions. In Experiment II, to confirm the role of soluble ST2 on depression-like behaviors, a depression test battery (two bottle choice test, forced swimming test, and tail suspension test) was applied. The general health check indicated good general health and normal gross appearance for ST2 Tg mice. Further, the neurological reflexes of all the mice were normal. We found that soluble ST2 overexpression resulted in decreased social interaction. Moreover, depression-like behaviors of ST2 Tg mice were observed in two well-established behavioral paradigms, the forced swimming test and the tail suspension test. Nevertheless, hedonic reaction to sucrose was observed in ST2 Tg mice similar to WT mice. These results suggest the depression in the ST2 Tg mice. In conclusion, through a series of experiments, we established the animal model for assessing role of soluble ST2 in neuropsychiatric disorders, and revealed the possible involvement of soluble ST2 in depressive behavior.

## Introduction

Psychoneuroimmunological studies have markedly accumulated knowledge about the interactions between the central nervous system and immune system. Especially, clinical and experimental evidences have clearly demonstrated that both cellular and humoral immunity are related to major depression [1]. For instance, the pro- and anti-inflammatory cytokines are disturbed in patients with major depression [2–4]. Experimental evidence also indicates that cytokines reach the brain through the blood-brain-barrier and/or bind to their receptors associated with the vagus nerve [5, 6].

*ST2* gene products mainly come in two forms: transmembrane (ST2L) and soluble secretory forms (ST2) [7–9]. ST2L was identified as the IL-33 receptor [8]. Today, it is understood that soluble ST2 binds to IL-33 and consequently interferes with the binding of IL-33 to ST2L [10]. Since the IL-33/ST2L pathway leads to MyD88, TRAF6-dependent activation of MAPK and NF-κB, and secretion of proinflammatory Th2-type cytokines [11, 12], soluble ST2 is regarded as a key molecule regulating immune system as well as cell proliferation. Indeed, soluble ST2 is reported to reduce IL-33-induced IL-6 and TNF-α production in macrophages and IL-33-induced IL-5 and IL-13 production in type 2 innate lymphoid cells [13, 14]. Previous studies revealed expression of *ST2* mRNA in mouse spleens, bone marrow, lymph nodes, and lungs [9]. ST2L immunoreactivity was also reported in several brain regions such as the olfactory bulb, anterior commissure, corpus callosum, hippocampus, somatosensory cortex, and motor cortex [15].

Pathophysiological roles of ST2 have been also investigated. Elevated serum concentrations of soluble ST2 were reported in patients with autoimmune diseases [16], asthma [17], myocardial infarction [18], idiopathic pulmonary fibrosis [19], and heart failure [20]. Furthermore, elevated serum concentrations of soluble ST2 have been reported in patients with neuropsychiatric disorders [21, 22], suggesting pathophysiological roles of soluble ST2 also in behavioral phenotypes. Clinical and experimental studies in psychoneuroimmunology have clearly demonstrated that both cellular and humoral immunity are related to major depression [1–4]. Nevertheless, the relation between soluble ST2 and depressive behavior remain to be uncovered. To complement this point, we performed broad behavioral phenotyping, utilizing transgenic mice with a high concentration of serum ST2 in the present study.

## Methods

### Animals

Soluble ST2 overexpression mice (ST2 Tg) were generated on a C3H/HeJ background [13]. At least twenty generations of ST2 Tg mice were crossed onto the BALB/c genetic background (Japan SLC Inc., Shizuoka, Japan) and maintained as heterozygote. ST2 transgene-negative and ST2 transgene-positive littermates were designated as WT and ST2 Tg mice, respectively. WT and ST2 Tg mice were housed in separate cages after 4 weeks of age. Mice were housed three to five per cage in a specific pathogen-free animal facility under controlled temperatures (23 ± 3 °C) and controlled light schedule (lights on from 7 A.M. to 7 P.M.), with food and water available ad libitum. Male mice at 8–11 weeks of age were used for the experiments.

All experimental procedures were performed in accordance with the National Institutes of Health Guidelines for the Care and Use of Laboratory Animals, under the animal protocols approved by the Institutional Animal Care and Use Committee of Jichi Medical University.

### Reverse transcription (RT)-PCR analysis

DNase I-treated total RNAs were isolated from indicated tissues, and subjected to RT-PCR analysis as described previously [10]. The PCR primers were as follows: ST2 (forward for exogenous and endogenous 5′-TGGCATGATAAGGCACACCATAAGGCT-3′, reverse for exogenous 5′-GCAGCCTGCACCTGAGGAGTGAA-3′, reverse for endogenous 5′-GTTAGTGTCTCTCTCCCTCCCATGC-3′), and β-actin (forward 5′-ATCTACGAGGGCTATGCTCT-3′, reverse 5′-TACTCCTGCTTGCTGATCCA-3′). The PCR products were separated on 2% agarose gels.

### Enzyme-linked immunosorbent assay (ELISA)

The concentration of soluble ST2 in serum was measured by a sandwich ELISA as described previously [23].

### Behavioral tests

Before beginning testings, each mouse was observed in a clean cage for a general health check and neurological screening tests according to methods of our previous reports [24, 25]. The neurological screening tests were designed to detect any gross abnormalities in physical function. The ear-twitch reflex occurred when the pinna was touched with a cotton swab from behind, resulting in immediate movement of the ear. The eye-blink reflex occurred when a cotton swab was approaching the eye, resulting in blinking. The postural reflex was evaluated by placing the mouse in an empty cage and shaking the cage, eliciting the extension of all four legs to keep an upright, balanced position. The righting reflex was tested by turning the mouse over onto its back, eliciting an immediate turnover response to restore the upright posture on all four feet. The whisker-touch reflex was tested by lightly touching whiskers of a freely moving mouse. Normal mice will stop moving their whiskers and turn the head to the side on which the whiskers were touched.

In Experiment I, comprehensive behavioral phenotyping was performed to reveal the role of soluble ST2 on sensorimotor functions, anxiety-like behaviors, a depression-like behavior, social behaviors, and learning and memory functions. In a visual placing test, a Preyer reflex test, a von Frey filament test, an olfactory habituation dishabituation test, a rotarod test, a wire hang test, a light-dark exploration test, a general activity monitoring, a novel place/object recognition test, social interaction test, and a social recognition test, the same mice were used (WT: *n* = 9, ST2 Tg: *n* = 9). In a hot-plate test and a forced swimming test, the same mice were used (WT: *n* = 9, ST2 Tg: *n* = 11). In a tube test, paired encounters of WT and ST2 Tg mice (WT: *n* = 10, ST2 Tg: *n* = 10) were staged based on body weight. In a novel place/object recognition test, social interaction test, and a social recognition test, the same mice were used (WT: *n* = 5, ST2 Tg: *n* = 5). In Experiment II, to confirm the role of soluble ST2 on depression-like behaviors and to check the reproducibility of result of the forced swimming test, a depression test battery (two bottle choice test, forced swimming test, and tail suspension test) was applied to the same mice (WT: *n* = 12, ST2 Tg: *n* = 12). The behavioral assessments were performed by experimenters blinded to the genotype. Information about the test order and time of day were provided in Supplementary Table 1.

### Experiment I: comprehensive behavioral phenotyping

#### Visual placing test

The visual placing test was used to assess the visual function [25]. Each mouse was suspended by the tail approximately 30 cm above a flat table surface and then gradually lowered to the Table. A normal mouse extends its forepaws for a “soft landing” as the table surface approaches. A blind mouse does not see the approaching surface so that the mouse does not extend its forelimbs until its whiskers or nose touch the table. Extension of the forepaws was recorded as a yes or no response by the investigator.

#### Preyer reflex test

The Preyer reflex test was used to assess the auditory function [25]. The Preyer reflex is a flinch response to the sound of a loud hand clap. The reflex was recorded as a yes or no response by the investigator.

#### Von Frey filament test

The von Frey filament test was used to assess tactile function [25]. Each mouse was placed on an elevated platform with wide gauge wire mesh surface. A von Frey hair (2.9 N) was inserted through the mesh holes from underneath to poke the undersurface of a hind paw. A normal response was defined as the mouse quickly flicking its paw away from the von Frey hair. If the mouse showed the normal responses in two out of three consecutive trials, its tactile ability was recorded as normal.

#### Olfactory habituation/dishabituation test

The olfactory habituation/dishabituation test was used to assess olfaction [25]. The odorant stimuli were tap water, vanilla extract (Kyoritsu-foods, Tokyo, Japan) diluted 1:100 in tap water, and rum extract (Kyoritsu-foods) diluted 1:100 in tap water. The odorant stimuli and the dilution factors were determined based on pilot experiments in our laboratory. Experimenters presented stimuli by dipping a cotton swab in the stimulus solution and then placing it through the wire lid of the cage. The cotton swab was positioned 4.4 cm from the bottom of the cage. Each stimulus was presented for 3 min and then replaced with a fresh swab scented with the same odorant for a total of three presentation of each stimulus and nine total olfactory stimulus presentations. The order of presentation was water (3x), vanilla (3x), and rum (3x). An experimenter recorded the cumulative time that the mouse spent in sniffing the cotton swab with a stopwatch. Sniffing was defined as (a) tilting the head upward with the nose oriented toward and within 2 cm of the swab, (b) rearing with the nose oriented toward and within 2 cm of the swab, and (c) physically contacting the muzzle to the swab if the mouth was closed. Occasional open-mouth contacts were considered to be chewing and not included in the cumulative sniff time.

#### Hot-plate test

Thermal pain sensitivity was assessed by a hot-plate test [26]. The test was performed according to the method described by our previous report [25]. The mouse was placed on a metal surface (19 cm in diameter, Muromachi Kikai, Tokyo, Japan) maintained at 54 ± 0.1 °C. The hot plate was surrounded by a transparent plastic barrier 20-cm in diameter and 25-cm in height. The latency to jumping off the plate or licking a hind paw was recorded. Sixty seconds was used as a cut-off time to protect the paw against injury.

#### Rotarod test

Motor coordination and balance were assessed using an accelerating rotarod (Muromachi Kikai Co., LTD) as described previously [25]. Mice were placed on a cylinder that slowly accelerated from 4 to 40 rpm the latency to fall recorded for a maximum of 300 s. When a mouse is placed on a rotating cylinder, the mouse must continuously walk forward to keep from falling off the rotarod. Each mouse performed three trials.

#### Wire hang test

Motor function was assessed using the wire hang test, which requires balance and grip strength [25]. A standard wire cage lid was used for this test. Masking tape placed around the perimeter of the lid prevented the mouse from walking off the edge. The test was performed by placing the mouse on the top of a wire cage lid. The investigator shook the lid lightly three times to cause the mouse to grip the wires and then turned the lid upside down. The upside-down lid was held approximately 40 cm above the cage litter, high enough to prevent the mouse from easily climbing down but not high enough to cause harm in the event of a fall. Each mouse performed two trials. The investigator used a stopwatch to time the latency in falling off the wire lid with a maximum of 60 s. The average of two trials was used as an indicator.

#### General activity monitoring

The general activity monitoring was performed using a cubical box (30 × 36 × 17 cm) made of white Plexiglas. Black lines were drawn on the floor with a marker. The lines divided the floor into nine 10 × 12 cm squares. The mouse was placed in the central square in the middle of the cubical box, and the number of line crossing was measured for 10 min under dim lighting (4 lx). A line-crossing was counted only when the animal crossed the line with all four paws.

#### Light-dark exploration test

The light-dark exploration test was performed as described previously [25]. The apparatus consisted of a polypropylene cage (44 × 21 × 21 cm) separated into two compartments by a partition with an aperture (12 × 5 cm) at floor level. The larger compartment (29 cm long) was open-topped, transparent, and brightly illuminated by white light from a 40 W desk lamp (1000 lx). The smaller compartment (15 cm long) was close-topped and painted black. Mice were placed individually in the center of the light compartment, facing away from the partition, and allowed to freely explore the apparatus for 10 min. The number of light-dark transitions between the two compartments and the total time spent in the dark compartment were recorded.

#### Forced swimming test

To evaluate depression-like behavior of mice, behavioral immobility termed “learned helplessness” was assessed by the Porsolt forced swimming test [27, 28]. The test was performed according to the method described in our previous report [25]. Each mouse was placed in individual glass cylinders (24.5 cm tall, 19 cm in diameter) filled with water (23–25 °C water) to a depth of 15 cm. The mice could not support themselves by placing their paws on the floor of the cylinder. Behavioral scoring employed a standard 6-min test duration. The duration of immobility during the last 4 min of the test period was quantified. A mouse was judged to be immobile when the mouse stopped swimming and remained floating with minimal movements necessary to keep its head above water.

#### Tube test

Social dominance was assessed by a tube test. The tube test assay was adapted from Lindzey et al. [29]. The test was performed according to the method described in our previous report [25]. The test employed a transparent Plexiglas tube 30 cm in length with a 3-cm inside diameter, which is sufficient to permit one adult mouse to pass through and to keep from reversing direction. For training, each mouse was released at alternating ends of the tube to run through the tube, sometimes with the help of a plastic stick pushing at its back. Each mouse was given eight training trials. Then, the mice were given the test trial. In the test trial, two mice were released simultaneously at opposite ends, and care was taken to ensure that they met in the middle of the tube. The mouse that first retreated from the tube within 2 min was designated the “loser”. In rare cases when no mouse retreated within 2 min, the tests were repeated. If the game was not resolved after two matches, a draw was made. Between each trial, the tube was cleaned with 70% ethanol. In WT mice, five WT mice (11 weeks of age) were cagemates and the other five WT mice (10 weeks of age) were cagemates. In ST2 Tg mice, five ST2 Tg mice (11 weeks of age) were cagemates and the other five ST2 Tg mice (10 weeks of age) were cagemates. Between the WT and ST2 Tg mice (WT *n* = 10, ST2 Tg n = 10), paired encounters were staged based on body weight and age not using a round-robin design. Those with similar weights competed.

#### Novel place/object recognition test

To evaluate learning ability of mice, recognition memory about place and object was assessed by a novel place/object recognition test [30]. The test was performed according to the method described in our previous report [25]. Briefly, the test was performed using a cubical open field (30 × 36 × 17 cm). The test consisted of three sessions with intertrial intervals of 2 min. During the habituation session, four different plastic objects were presented in open field. Exploration of four different plastic objects in open field was measured for 15 min under dim lighting (4 lx). For the place recognition session, the four objects initially placed in a square arrangement were reconfigured into a polygon-shaped pattern by moving two objects (displaced objects, DOs). The remaining two objects were left at the same location (non-displaced objects, NDOs). The time spent exploring the DOs and NDOs was recorded for 5 min. In the object recognition session, one of the NDOs was replaced with a novel object (NO), and the two DOs were removed. The time examining the NO or familiar NDO was recorded for 5 min. Exploratory behavior was defined as (a) tilting the head upward with the nose oriented toward and within 2 cm of the object and (b) physically contacting the muzzle or forelimb to the object. The percentage of novelty preference was calculated as follows: (novel place or object exploration duration) / (novel place or object exploration duration + familiar place or object exploration duration) × 100.

#### Social interaction test and social recognition test

To examine social interest to a same-sex BALB/c mouse (8–11 weeks of age), each mouse was allowed to explore an unfamiliar male mouse in a cubical box (30 × 36 × 17 cm) for 10 min. Two identical, wire cup-like containers with removable lids large enough to hold a single mouse were used. These were placed vertically inside the apparatus, one in each side chamber, and contained a naïve mouse or not. Each container was comprised of metal wires to allow for air exchange between the interior and exterior of the cylinder but small enough to prevent direct physical interactions between the inside animal and outside. Time spent exploring the mouse was recorded. Exploratory behavior was defined as (a) tilting the head upward with the nose oriented toward and within 2 cm of the mouse and (b) physically contacting the muzzle or forelimb to the mouse.

Recognition memory of other individuals was assessed by a social recognition test [25]. One hour later following the social interaction test, the mouse was returned to the cubical box and exposed to the mouse encountered at the social interaction test and a novel mouse for 10 min. Two identical, wire cup-like containers were also used. These contained the same, or novel mouse. Time spent exploring each mouse was recorded. The percentage of novelty preference was calculated as follows: (novel individual exploration duration) / (novel individual exploration duration + familiar individual exploration duration) × 100.

#### Experiment II: depression test battery

Depression-like behavior of mice were assessed by the following three tests: (1) Two bottle choice test was performed to examine hedonic reaction to sucrose [24]. The test was performed according to the method described in our previous report [24]. Briefly, the mouse was placed in a square plastic cage and habituated for approximately 24 h. During this habituation period, the mouse was allowed to drink from two bottles containing water. After the habituation period, one of the two bottles was replaced with a bottle containing 5% sucrose and consumption monitored for 24 h. The preference for sucrose was calculated using the following formula: Preference (%) = sucrose consumption (g) / total consumption (g) × 100. Water and food consumption were also measured during this habituation period. After 24 h from the onset of the habituation procedure, the mouse was briefly removed from its cage and weighed, and the amount of food remaining including any on the bottom of the cage was recorded using an electronic analytical scale (EK-610i, A&D Company, Tokyo, Japan). Food consumption was calculated as the difference between the amount of food at the start and at the end of habituation period. The two bottles containing water were also weighed using the same electronic analytical scale at the onset and end of the habituation period to calculate the water consumption. The water and food consumption were calculated using the following formula: Water or food consumption (g/g) = water or food consumption (g) / body weight (g). (2) Antidepressant activity was assessed by the Porsolt forced swimming test [27, 28]. (3) Antidepressant activity was also assessed by the tail suspension test [31, 32]. Briefly, mice were securely fastened to a flat metallic surface by the tip of the tail using medical adhesive tape and suspended 30 cm above the ground. The time of immobility was quantified.

### Statistical analysis

The differences in the mean between two groups were tested using Welch’s t-test after transformation to ranks. Two-way ANOVA with repeated measures was used to assess tests containing two variables. A chi-square analysis was used for tube test. *P*-values less than 0.05 were regarded as statistically significant.

## Results

### Expression of exogenous and endogenous soluble ST2 mRNAs

**Fig. 1:**
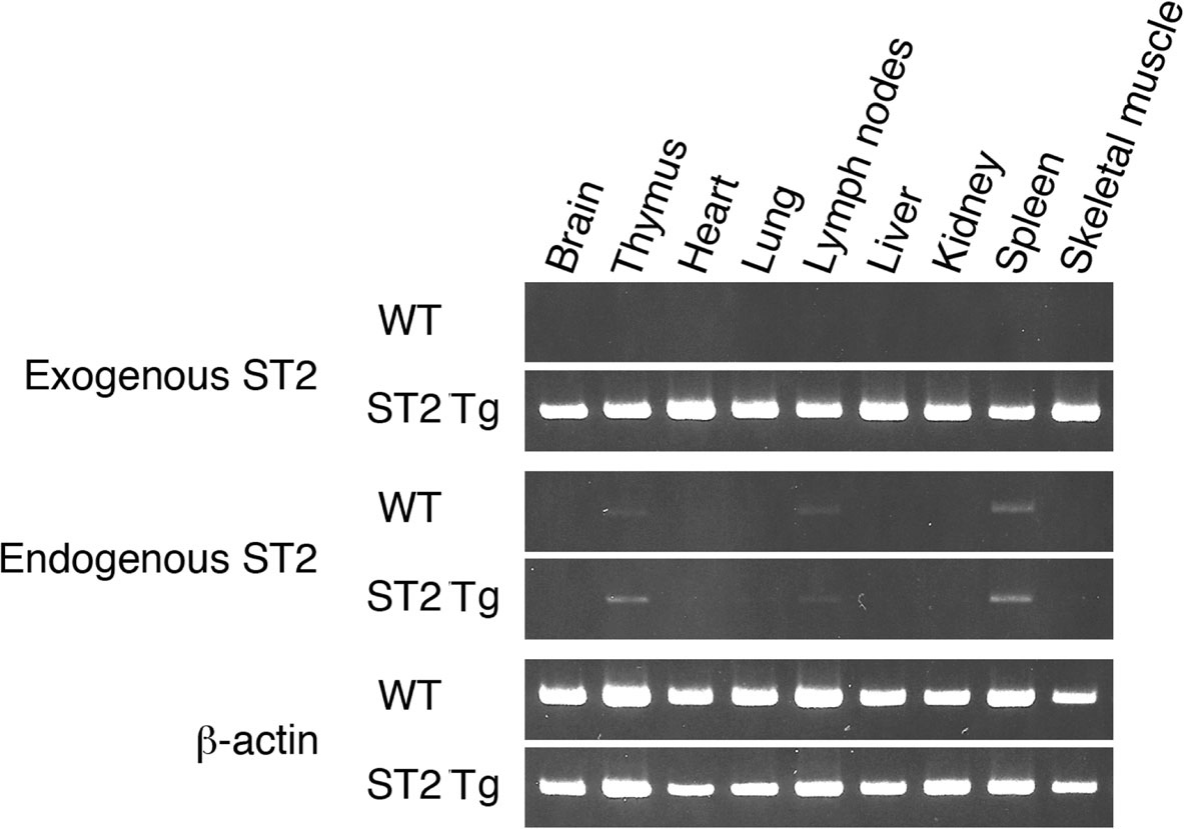
Expression of exogenous and endogenous soluble ST2 mRNAs. RT-PCR was performed on mRNAs derived from indicated tissues in untreated WT and ST2 Tg mice. β-actin was used as an internal endogenous control. The size of PCR products was as follows: exogenous ST2 (596 bp), endogenous ST2 (754 bp), and β-actin (576 bp)

To ascertain the expression of soluble ST2 mRNA in untreated WT and ST2 Tg mice, we performed an RT-PCR analysis (Fig. 1). Exogenous soluble ST2 mRNA derived from the transgene was expressed in all examined tissues of ST2 Tg mice. On the other hand, endogenous soluble ST2 mRNA was slightly expressed in the thymus, lymph nodes, and spleen of both WT and ST2 Tg mice. In addition, serum level of soluble ST2 in ST2 Tg mice was significantly higher than that in WT mice (Table 1, t = 5.773, df = 7.200, *p* < 0.001).

**Table 1:** Serum levels of soluble ST2 in WT and ST2 Tg mice

Group	WT			ST2 Tg		
Serum ST2 (ng/mL)	1.9	±	1.6	287.6	±	44.3***

The concentration of soluble ST2 was measured by sandwich ELISA (WT: n = 5, ST2 Tg: *n* = 5). Values are mean ± SEM. *** *p* < 0.001 vs. WT

### General health

The general health check indicated good general health and normal gross appearance for the WT and ST2 Tg mice (Table 2). The neurological reflexes of all the mice were normal (Table 2).

**Table 2:** General health and neurological reflexes

Group	WT			ST2 Tg		
General health check						
Bald patch (% with)	0.0	±	0.0	0.0	±	0.0
Body size (% with average size)	100.0	±	0.0	100.0	±	0.0
Body weight (g)	26.4	±	0.4	26.2	±	0.3
Crustiness around the nostrils/eyes (% with)	0.0	±	0.0	0.0	±	0.0
Ear pinna/Footpad color (% with normal color)	100.0	±	0.0	100.0	±	0.0
Fur (% with normal fur)	100.0	±	0.0	100.0	±	0.0
Gait (% with normal gate)	100.0	±	0.0	100.0	±	0.0
Leisons on the feet/tail (% with)	0.0	±	0.0	0.0	±	0.0
Posture (% with normal posture)	100.0	±	0.0	100.0	±	0.0
Scabs on the tail, rump, back (% with)	0.0	±	0.0	10.0	±	10.0
Tumor (% with)	0.0	±	0.0	0.0	±	0.0
Whisker (% with normal whisker)	97.8	±	2.2	95.7	±	3.0
Neurological screening test						
Ear twich (% with quick response)	100.0	±	0.0	100.0	±	0.0
Eye blink (% with normal response)	100.0	±	0.0	100.0	±	0.0
Postual reflex (% with normal response)	100.0	±	0.0	100.0	±	0.0
Righting reflex (% with normal response)	100.0	±	0.0	100.0	±	0.0
Whisker-touch (% with normal response)	95.6	±	3.1	93.6	±	3.7

The general health check and neurological screening test indicated good general health, normal gross appearance, and normal neurological reflexes of all the mice (WT: *n* = 45, ST2 Tg: *n* = 47). Values are mean ± SEM

### Sensory functions

The results of the visual placing test, Preyer reflex, and Von Frey hairs touch test are provided in Table 3. The visual placing test indicated normal visual function in all mice, and the Preyer reflex was also similar between the two groups. Moreover, the Von Frey hairs touch test indicated normal tactile function.

**Table 3:** Sensory functions

Group	WT			ST2 Tg		
Visual placing test (% with normal response)	100.0	±	0.0	100.0	±	0.0
Preyer reflex test (% with normal response)	100.0	±	0.0	100.0	±	0.0
Von Frey filament test (% with normal response)	89.0	±	11.0	100.0	±	0.0

No significant differences were observed between the two groups. The visual placing test, Preyer reflex test, and Von Frey hairs touch test indicated normal visual, auditory, and tactile function in all mice (WT: *n* = 9, ST2 Tg: *n* = 9). Values are mean ± SEM

**Fig. 2:**
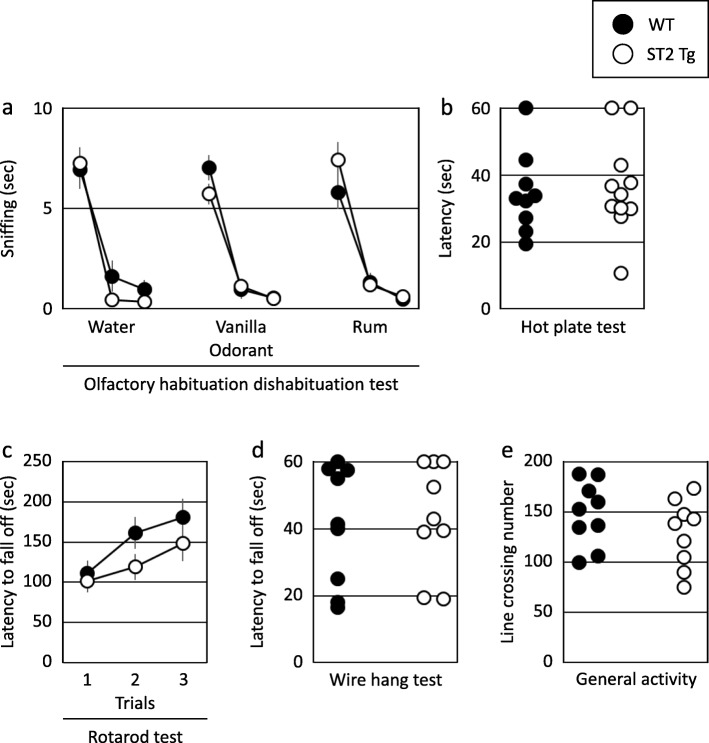
Sensorimotor functions of ST2 Tg mice. **a** The cumulative time spent in sniffing water, vanilla or rum scents (WT: *n* = 9, ST2 Tg: *n* = 9). **b** The time required before jumping off a hot plate or licking a hind paw in the hot plate test (WT: *n* = 9, ST2 Tg: *n* = 11). **c** The length of time before falling off the rotarod (WT: *n* = 9, ST2 Tg: *n* = 9). **d** The mean latency for falling off the wire in the wire hand test (WT: *n* = 9, ST2 Tg: *n* = 9). **e** The number of line crossing in general activity monitoring (WT: *n* = 9, ST2 Tg: *n* = 9)

The mean cumulative time spent on sniffing water, vanilla, or rum in the olfactory habituation/dishabituation test is shown in Fig. 2a. Two-way ANOVA with repeated measures revealed a significant main effect of odor (F = 57.725, df = 1, 8, *p* < 0.001). No significant effects of group (F = 0.179, df = 1, 8, *p* = 0.672) or the interaction of group × odor (F = 1.291, df = 1, 8, *p* = 0.252) were identified. Normal olfactory function was observed in all mice.

The WT and ST2 Tg mice showed a similar pain response to a hot plate. No significant difference was observed (Fig. 2b, t = 0.408, df = 17.113, *p* = 0.687).

### Motor functions

The mean latency for falling off the rod in the rotarod test is shown in Fig. 2c. Two-way ANOVA with repeated measures revealed a significant main effect of trial (F = 4.809, df = 1, 2, *p* = 0.012). No significant effects of group (F = 3.395, df = 1, 2, *p* = 0.071) or the interaction of group × trial (F = 0.378, df = 1, 2, *p* = 0.686) were identified. The rotarod test indicated normal motor coordination and motor learning function in all mice.

The mean latency for falling off the wire in the wire hang test is shown in Fig. 2d. The wire hang test indicated normal balance and grip strength in all mice (t = 0.433, df = 15.999, *p* = 0.670).

The number of line crossing in general activity monitoring was similar between the two groups (Fig. 2e t = 1.111, df = 15.988, *p* = 0.282).

### Anxiety- and depression-like behaviors

**Fig. 3:**
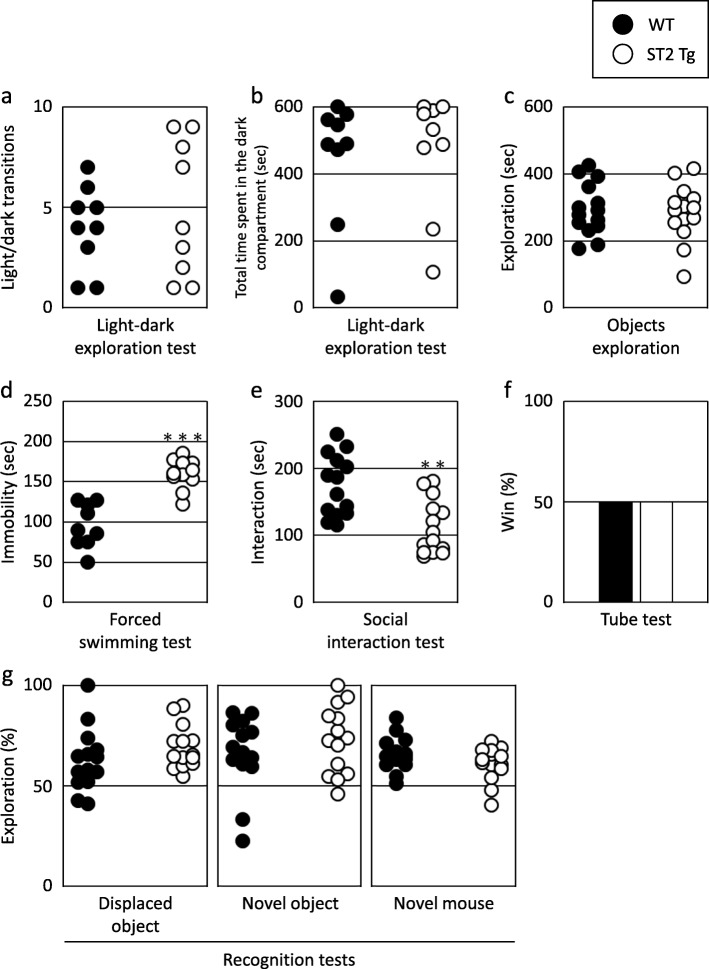
Behavioral characteristics of ST2 Tg mice. **a** The total number of transitions between chambers in the light-dark exploration test (WT: *n* = 9, ST2 Tg: *n* = 9). **b** The time spent in the dark compartment in the light-dark exploration test (WT: *n* = 9, ST2 Tg: *n* = 9). **c** The exploration time during the habituation period of the novel place/object recognition test (WT: *n* = 14, ST2 Tg: *n* = 14). **d** The time of immobility during the last 4 min of the Porsolt forced swimming test (WT: *n* = 9, ST2 Tg: *n* = 11). **e** The interaction time in the social interaction test (during the encoding phase of social recognition test) (WT: *n* = 14, ST2 Tg: *n* = 14). **f** The total percentage of wins of each animal group during the tube test (WT: *n* = 10, ST2 Tg: *n* = 10). **g** The percentage of exploration time for displaced objects, novel objects, or a novel mouse (WT: *n* = 14, ST2 Tg: *n* = 14). * statistically significant vs the ST2 Tg mice. ** *p* < 0.01, *** *p* < 0.001 statistically significant vs the WT mice

The total number of transitions between chambers and the time spent in the dark compartment in the light-dark exploration test are shown in Fig. 3a and b. No significant differences were observed in the total number of transitions (Fig. 3a, t = 0.478, df = 14.140, *p* = 0.639) and the total time spent in the dark compartment (Fig. 3b, t = 0.475, df = 15.581, *p* = 0.641). Furthermore, the WT and ST2 Tg mice showed similar exploration times during the habituation period of the novel place/object recognition test (Fig. 3c, t = 0.090, df = 25.984, *p* = 0.928). These results suggest that two groups of mice showed similar anxiety-like behavior.

Behavioral depression was assessed using the forced swimming test. The immobility time during the forced swimming test was significantly longer in ST2 Tg mice compared to the WT mice (Fig. 3d, t = 6.377, df = 17.973, *p* < 0.001).

### Social behaviors

In the social interaction test, each mouse was allowed to explore in an open field with a naïve male mouse to assess social interest in an unfamiliar mouse. The WT mice showed longer total interaction times (Fig. 3e, t = 3.664, df = 25.907, *p* = 0.001).

Social dominance/aggressiveness was assessed using a tube test. The total winning percentages of the WT and ST2 Tg mice were similar (Fig. 3f, χ^2^ = 0, df = 1, *p* = 1). WT mice won three times and drew four times. The ST2 Tg mice, on the other hand, won three times only.

### Learning and memory functions

In the novel place/object recognition test, the WT and ST2 Tg mice showed similar longer exploration times for displaced than non-displaced objects (Fig. 3g, Displaced object: t = 1.352, df = 24.123, *p* = 0.188). Further, no significant differences were observed in the exploration times for the novel object (Fig. 3g, Novel object: t = 0.590, df = 25.243, *p* = 0.560). In the social recognition test, the WT and ST2 Tg mice showed similar longer interaction times with the novel mouse (Fig. 3g, Novel mouse: t = 1.107, df = 25.998, *p* = 0.278).

### Experiment II: depression-like behaviors

**Fig. 4:**
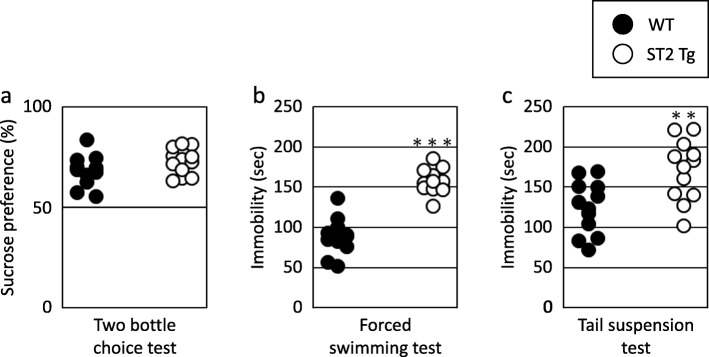
Depression-like behaviors in ST2 Tg mice. **a** The preference ratio for 5% sucrose to water in the two bottle choice test (WT: *n* = 12, ST2 Tg: *n* = 12). **b** The time of immobility during the last 4 min of the Porsolt forced swimming test (WT: *n* = 12, ST2 Tg: *n* = 12). **c** The time of immobility during the tail suspension test (WT: *n* = 12, ST2 Tg: *n* = 12). ** p < 0.01, *** p < 0.001 statistically significant vs the WT mice

To examine sucrose preferences, the mice were allowed to drink water or 5% sucrose bottle. Both the WT and ST2 Tg mice preferred sucrose to water, whereas no significant difference was observed between the groups (Fig. 4a, t = 1.545, df = 21.999, *p* = 0.136). Both the WT and ST2 Tg mice also showed similar water (Table 4, t = 0.147, df = 15.671, *p* = 0.884) and food consumption (Table 4, t = 0.221, df = 16.499, *p* = 0.827).

**Table 4:** Water and food consumption

Group	WT			ST2 Tg		
Water consumption/ BW (g/g)	0.54	±	0.02	0.55	±	0.04
Food consumption/ BW (g/g)	0.15	±	0.01	0.15	±	0.02

No significant differences were observed between the two groups (WT: *n* = 12, ST2 Tg: *n* = 12). Values are mean ± SEM

Behavioral depression was assessed using the forced swimming test and the tail suspension test. The immobility time during the forced swimming test was significantly longer in ST2 Tg mice compared to WT mice (Fig. 4b, t = 7.723, df = 22.000, *p* < 0.001). The immobility time during the tail suspension test was also longer in ST2 Tg mice compared to the WT mice (Fig. 4c, t = 3.218, df = 21.493, *p* = 0.004).

## Discussion

In this study, we report a possible role of the soluble ST2 in the social and depression-like behaviors in mice. We found that soluble ST2 overexpression resulted in decreased social interaction. Moreover, depression-like behaviors of ST2 Tg mice were observed in two well-established behavioral paradigms, the forced swimming test and the tail suspension test. Nevertheless, hedonic reaction to sucrose was observed in ST2 Tg mice similar to WT mice. These results suggest the depression in the ST2 Tg mice.

It is reported that soluble ST2 binds to IL-33, and consequently interferes the binding of IL-33 to ST2L [10]. Hence, it is hypothesized that IL-33 or ST2L abnormality affects behavioral trait in same behavioral categories: social behavior and depression-like behavior. Dohi et al. [33] reported that IL-33 knockout mice on a C57BL/6 J background showed impaired social recognition in the three-chamber social interaction test. In addition, IL-33 deficiency caused anxiolytic effects in mice assessed by elevated plus maze test and open field test. A possible explanation may be that soluble ST2 overexpression mimics IL-33 deficiency, because soluble ST2 interferes the binding of IL-33 to ST2L. Nevertheless, soluble ST2 overexpression did not cause antidepressant effects in our study. Moreover, ST2 Tg mice did not show impaired social recognition memory.

There are two possible explanations for the discrepancy between the results of these two studies. One possibility is differences in genetic background cause different behavioral phenotypes. Since we used ST2 Tg mice on a BALB/c background strain, and not C57BL/6 J background strain, the direction of effects to behavioral phenotypes in the disturbance of the IL-33/ST2L pathway may depend on background strain. In support of this hypothesis, the previous study reported the impact of genetic background on behavior and neurodegeneration, which closely related to immune system in mice [34]. The other possibility is that the methodological differences lead to different results. There were methodological differences in test protocols between our study and Dohi et al. For example, we used the light-dark box test for measuring anxiety-like behavior, although Dohi et al. performed the elevated plus-maze test. Furthermore, since we did not confirm whether the reduction in IL-33 occurred in ST2 Tg mice, care should be taken in interpreting this result.

How did soluble ST2 and IL-33/ST2L affect behavioral phenotypes in mice? Since we have confirmed elevated soluble *ST2* mRNA expression in brain, thymus, heart, lung, lymph node, liver, kidney, spleen, and skeletal muscle in our ST2 transgenic mice, which also showed high concentration of soluble ST2 in serum, two possibilities can be considered: 1) Soluble ST2 in the brain directly affects the behaviors and/or 2) Soluble ST2 outside of the brain causes the behavioral changes. Although the level of soluble ST2 in the serum of WT mice was below the ELISA assay detection threshold, it is possible that a subthreshold level of soluble ST2 circulates in the blood and possibly reaches the brain to influence its function and behavior. Moreover, soluble ST2 overexpression in peripheral organs may affect the brain via another mediator such as cytokines, metabolites, and neural connections. IL-33 regulates gut microbiota homeostasis by promoting IgA production [35]. Germ-free mice, which lack gut microbiota, exhibited abnormality in emotional behavior [36–38]. Taken together, soluble ST2 overexpression may decrease social behavior and enhance depressive-like behavior by altering the composition of gut microbiota. Future studies on brain-specific overexpression of the soluble ST2 gene will elucidate influence of central and peripheral soluble ST2 to behaviors.

We are aware that the present study does have some limitations. The overexpression of soluble ST2 did not fall within the normal physiological fluctuation. Indeed, ST2 Tg mice showed three hundredfold increase in serum soluble ST2 concentration. Therefore, although this is the first report to systematically investigate the behavioral phenotypes of soluble ST2 overexpressing mice, further studies are required to confirm our hypotheses. Our finding may have clinical utility. In fact, increased soluble ST2 in serum of AD patients was reported [21]: IL-33 reduces β-amyloid levels and amyloid plaques and reverses synaptic plasticity impairment and memory deficit in a mouse model [21]. Moreover, serum levels of IL-33 and soluble ST2 positively correlate with cognitive performance in schizophrenia patients [22].

In conclusion, through a series of experiments we established the animal model for assessing role of soluble ST2 in neuropsychiatric disorder, and revealed the possible involvement of soluble ST2 in depressive behavior.

## Supplementary information

**Additional file 1.**

## Data Availability

The datasets supporting the conclusions of this article are included within the manuscript.

## Abbreviations

DOs: Displaced objects
NDOs: Non-displaced objects
NO: Novel object
